# Genotyping by multiplexed sequencing (GMS): A customizable platform for genomic selection

**DOI:** 10.1371/journal.pone.0229207

**Published:** 2020-05-01

**Authors:** Travis M. Ruff, Elliott J. Marston, Jonathan D. Eagle, Sajal R. Sthapit, Marcus A. Hooker, Daniel Z. Skinner, Deven R. See

**Affiliations:** 1 Department of Crop and Soil Sciences, Washington State University, Pullman, Washington, United States of America; 2 USDA-ARS Wheat Health, Genetics and Quality Research Unit, Pullman, Washington, United States of America; 3 Department of Plant Pathology, Washington State University, Pullman, Washington, United States of America; Institute of Genetics and Developmental Biology Chinese Academy of Sciences, CHINA

## Abstract

As genotyping technologies continue to evolve, so have their throughput and multiplexing capabilities. In this study, we demonstrate a new PCR-based genotyping technology that multiplexes thousands of single nucleotide polymorphism (SNP) markers with high-throughput capabilities in a simple protocol using a two-step PCR approach. The bioinformatic pipeline is user friendly and yields results that are intuitive to interpret. This method was tested on two recombinant inbred line (RIL) populations that had previous genotyping data from the Illumina Infinium assay for *Triticum aestivum* L. and the two data sets were found to be 100% in agreement. The genotyping by multiplexed sequencing (GMS) protocol multiplexes 1,656 wheat SNP markers, 207 syntenic barley SNP markers, and 49 known informative markers, which generate a possible 2,433 data points (including homoeoalleles and paralogs). This genotyping approach has the flexibility of being sequenced on either the Ion Torrent or Illumina next generation sequencing (NGS) platforms. Products are the result of direct sequencing and are therefore more reliable than scatter plot analysis which is the output of other genotyping methods such as the Illumina Infinium assay, komeptitive allele specific PCR and other like technologies.

## Introduction

As next generation sequencing (NGS) technologies continue to advance, single nucleotide polymorphisms (SNPs) have become the marker of choice for genotyping studies. SNPs are the most abundant source of genetic variability in eukaryotic organisms, are co-dominant and the most used marker systems for genetic studies [1–7]. SNPs are ubiquitous throughout genomes but are more often found in non-genic regions typically not affecting phenotypes [1]. Non-synonymous SNPs are of great value to researchers and breeders as they directly impact an organism’s phenotype [8,9]. Over the last 20 years geneticists have shifted from utilizing microsatellite marker sets and made a great effort in discovering SNPs and have developed them into markers. These SNP markers track both non-functional and functional SNPs but typically resulted in uniplex assays [2,10–13] which limited the amount of data generated per assay.

Current SNP-based genotyping technologies have allowed genetic studies to move from mostly uniplex assays; simple sequence repeats (SSRs), cleaved amplified polymorphic sequences (CAPS), kompetivite allele specific PCR (KASP) (LGC Genomics, Beverly, MA), and others; to high-throughput genotyping with high-density SNP panels [10,14,15]. Technologies such as the Illumina Infinium assays [16], genotyping-by-sequencing (GBS) [17,18] and other methods have allowed researchers to genotype hundreds of samples with thousands to millions of SNPs in a single assay [15]. These technologies have not only increased the speed of genotyping and overall data output, but have also decreased the cost of genotyping samples [9,10,13,15]. As much progress as these technologies have made, each has limitations.

The Infinium Assay from Illumina (Illumina, San Diego, CA) has achieved high-density genotyping with their hybridization-based SNP arrays. Limitations with this genotyping technology include its higher costs compared to other methods especially if a custom assay needs to be developed and data output can be difficult to interpret [13]. In polyploids, homozygous samples are commonly grouped into the heterozygous cluster which leads to the loss of the heterozygous samples. GBS is a more recent genotyping method which reduces genetic complexity, discovers variants across a genome, and genetically characterizes samples [7,17]. The major issues with GBS include the following: the random nature of the process, data analysis is bioinformatically challenging, discovered markers are principally dominant and results have a large amount of missing data which requires imputation [13]. Even though these and other genotyping methods continue to be used in wheat genotyping studies, a new method is needed. An ideal method would genetically characterize samples by targeting specific loci, be easily customized, high-density, high-throughput, and provide results that are easily interpreted.

To meet the need for a more targeted approach, a method that is PCR based would be ideal. This necessity is punctuated by multiple entities having recently developed genotyping methods that are PCR based, including Illumina’s partnership with ThermoFisher with their AmpliSeq technology (ThermoFisher Scientific, Waltham, MA) and Integrated DNA Technologies with their rhAMPSeq (Integrated DNA Technologies, Coralville, IA). In addition, various publications have detailed additional genotyping methods that are PCR based. Targeted Amplicon Sequencing (TAS) [3] is a genotyping method that uses a two-step PCR protocol that multiplexed 6 primer pairs for genotyping. These amplicons were sequenced on a Roche 454 NGS platform (Roche, Basel, Switzerland) and the data were analyzed using BarcodeCruncher [3] and Genome Sequence Data Analysis Software (Roche, Basel, Switzerland). Genotyping-in-Thousands by sequencing (GT-seq) [4] is also a two-step PCR protocol that multiplexed 192 SNP markers. GT-seq libraries were sequenced on an Illumina HiSeq 1500 and data were analyzed with a custom Perl script. Genotyping by Multiplexing Amplicon Sequencing in wheat (GBMAS) [5,10] is again a two-step PCR protocol that multiplexed 33 SNP markers. GBMAS libraries were sequenced on the Ion Proton and data analysis was done utilizing custom Perl scripts along with FLEXBAR [19] and BLAT [20] software. Three-round multiplex PCR for SNP genotyping with NGS [21] utilized a protocol with three PCR steps to generate an equivalent amount of PCR product from each primer pair from each sample which multiplexed 37 markers. Data analysis was accomplished by using custom Perl scripts, FASTX-Toolkit [22], samtools [23] and BWA [24] software. Multiplex PCR Targeted Amplicon Sequencing (MTA-Seq) [6] protocol used one round of PCR to generate 443 amplicons with targeted SNPs. These amplicons underwent an end repair for adaptor ligation, followed by a nick repair reaction and the resulting libraries were sequenced on an Ion Proton NGS platform. Sequence data were mapped using the Torrent Mapping Alignment Program (Thermo Fisher Scientific, Waltham, MA) and SNPs were determined with VarScan software [25]. These published methods meet some or most of the ideal PCR-based genotyping characteristics but are lacking in at least one of these areas, specifically marker depth.

In this study, we present a PCR-based genotyping technology that highly multiplexes SNP markers, is capable of being high-throughput, is easily customized to fit various genotyping studies, generates libraries that can be sequenced on either the Illumina or Ion Torrent NGS platforms and generates data that is simple to interpret. The genotyping by multiplexed sequencing (GMS) protocol employs 1,656 wheat SNP markers and 207 syntenic barley SNP markers [26] in 6 primer pools with ~370 primer pairs per pool. In addition to this marker set are 49 known informative makers (KIMs) which have been valuable in marker-assisted selection (MAS) in the hexaploid *Tricticum aestivum* L., common wheat.

## Results

### SNP panel metrics

The GMS protocol has a total of 5 wheat SNP primer pools and 1 syntenic barley primer pool, each containing ~370 primer pairs per pool. The KIMs added 49 more primer pairs for a total of 1,912 markers. This marker panel also amplified an additional 396 homoeoalleles and 125 paralogs which were assigned to specific chromosomes. The GMS SNP panel averaged ~104 markers per chromosome, the distance between markers on all chromosomes averaged 2.68 cM, with 41.63 cM being the largest gap on any chromosome (Table 1). GMS SNP primers generated data that was found to be in 100% agreement with the 90K SNP chip data from the two RIL populations.

**Table 1:** Overview of current wheat SNP panel metrics per chromosome.

Chrm.	Number of markers per chrm.	Avg. cM dist. per chrm.	Largest distance between markers (cM)
1A	94	1.77	13.73
1B	136	1.77	12.83
1D	77	4.09	25.96
2A	134	1.84	9.33
2B	132	1.93	9.68
2D	84	3.12	25.71
3A	89	2.38	11.03
3B	133	1.66	9.22
3D	36	4.49	17.75
4A	101	1.83	8.91
4B	91	1.64	13.98
4D	52	5.49	41.63
5A	150	1.25	10.63
5B	180	1.57	12.69
5D	100	3.73	37.89
6A	109	2.08	10.24
6B	101	1.59	6.55
6D	59	5.05	23.47
7A	121	2.59	14.83
7B	114	2.14	11.99
7D	84	4.31	25.27
Total number of wheat SNP results		2,177	
Avg. number of markers per chrm.		103.67	
Overall avg. distance between markers		2.68	

### KIMs

The KIMs were adapted from KASP assays that are used in wheat MAS that track important agronomic, quality and disease resistance traits. Primers were designed to incorporate the SNPs tracked by these assays and extend the length of generated products when needed. The KIM primer pool was run with the diversity panel (DP) and data was analyzed. To verify each marker had amplified the appropriate SNP, the DP sequence data was compared with each KASP markers sequence. This was accomplished by following Rasheed et al. [15] who described and validated KASP assays used in wheat MAS. After data analysis, of the 58 KASP markers converted to the GMS platform, 49 markers produced data.

### Barley markers

The current GMS barley SNP panel consists of 1,609 markers and were adapted from adapted the 50K barley SNP chip developed by Bayer et al. [26]. These barley markers were used to genotype the two wheat RIL populations. After data analysis of the sequencing run, 207 or 12.9% of barley markers generated SNP data in wheat.

### PCR optimization

**Fig 1:**
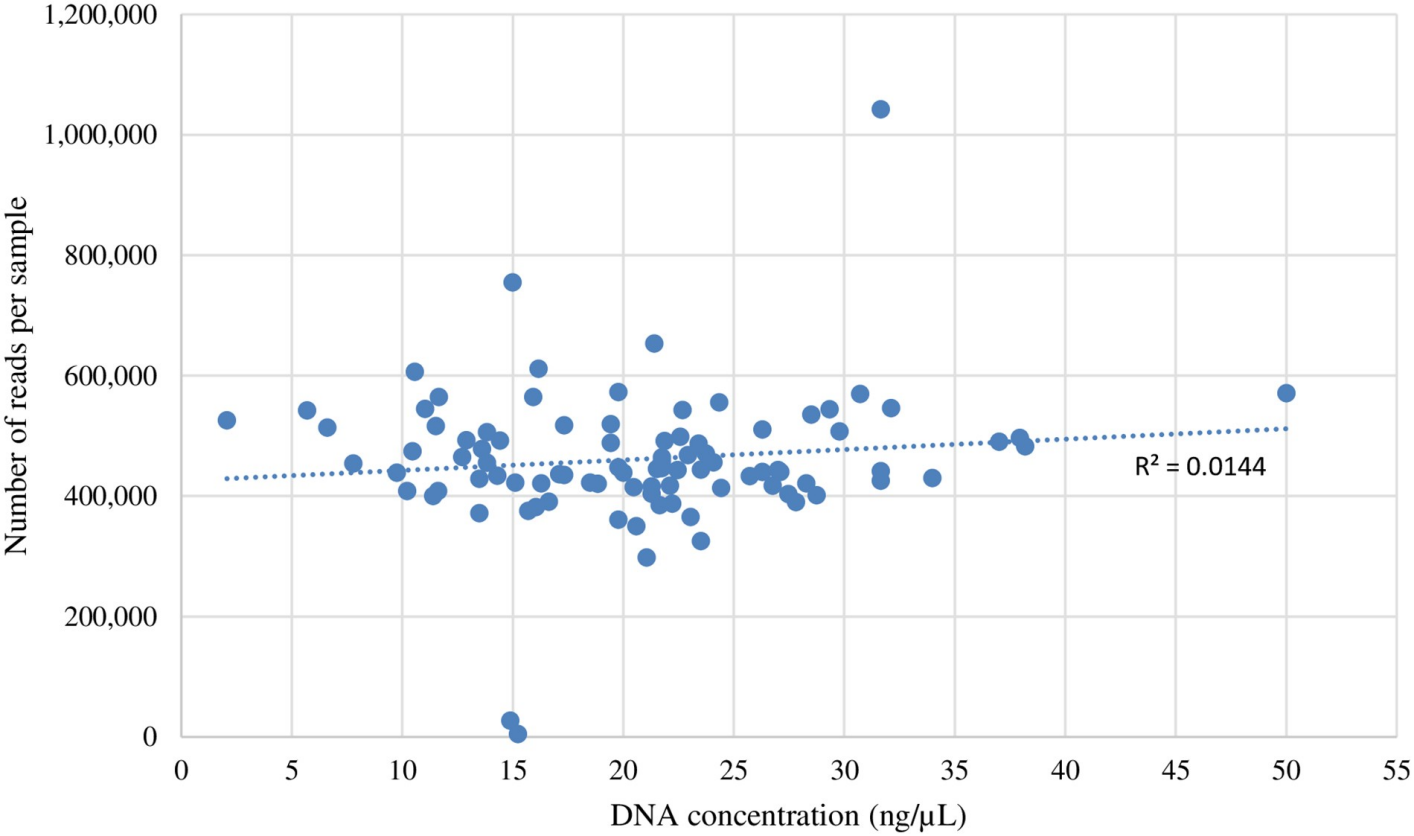
DNA concentrations vs reads per sample. Number of reads generated per sample (Y axis) from sample DNA concentrations in ng/μL (X axis).

Following the GMS protocol sample DNA plates were normalized to approximately 20 ng/μL before PCR #1, in which a total of 80 ng of template DNA was used per PCR reaction. DNA concentrations of the RIL sample plate ranged from 2.1 to 50.1 ng/μL. The average concentration of a normalized well was ~19.49 ng/μL with a standard deviation of 7.98 ng/μL. In the GMS runs of the RIL samples, one reaction had ~8 ng of total template DNA which generated 525,914 reads, while another reaction had ~200.4 ng of total template DNA which generated 570,664 reads (Fig 1).

Barcodes in PCR #2 were doubled to a concentration of 0.8 μM and were run with the RIL populations. After data analysis, this concentration of barcodes increased the number of sequence reads generated by 32.4%. The original barcode concentration was then tripled to 1.2 mM and run with the RIL populations. The data were analyzed and found no significant increase in the number of sequence reads that were generated. The GMS protocol continues to use the barcode concentration of 0.8 μM in PCR #2.

### Primer pool optimization

To determine the amplification rate of primer pairs within primer pools, the average number of reads per marker was calculated from five GMS runs and analyzed. The average reads per marker were grouped at 25 reads per marker and a histogram was created that consisted of 11 bins in total. This histogram revealed that 528 or 39.1% of all GMS markers yielded 25 reads per marker or less and averaged less than 1 read per marker in the first bin. The remaining 60.9% of markers generated at least 25 hits per marker or greater. To optimize the primer pools, they were reconstructed according to each marker’s rate of amplification. For example, the first bin was divided into two separate primer pools. The first reconstructed primer pool contained primer pairs that averaged less than one read per sample/run and consisted of 387 markers. The second half of bin one was combined with bin two to create the second reconstructed primer pool. This method of reconstructing primer pools continued until 8 new wheat primer pools were created. Primer pools 6–8 contained the best-performing primer pairs and each were combined with primer pool 5 at lesser concentrations to reduce the number of reads generated by these pools.

**Fig 2:**
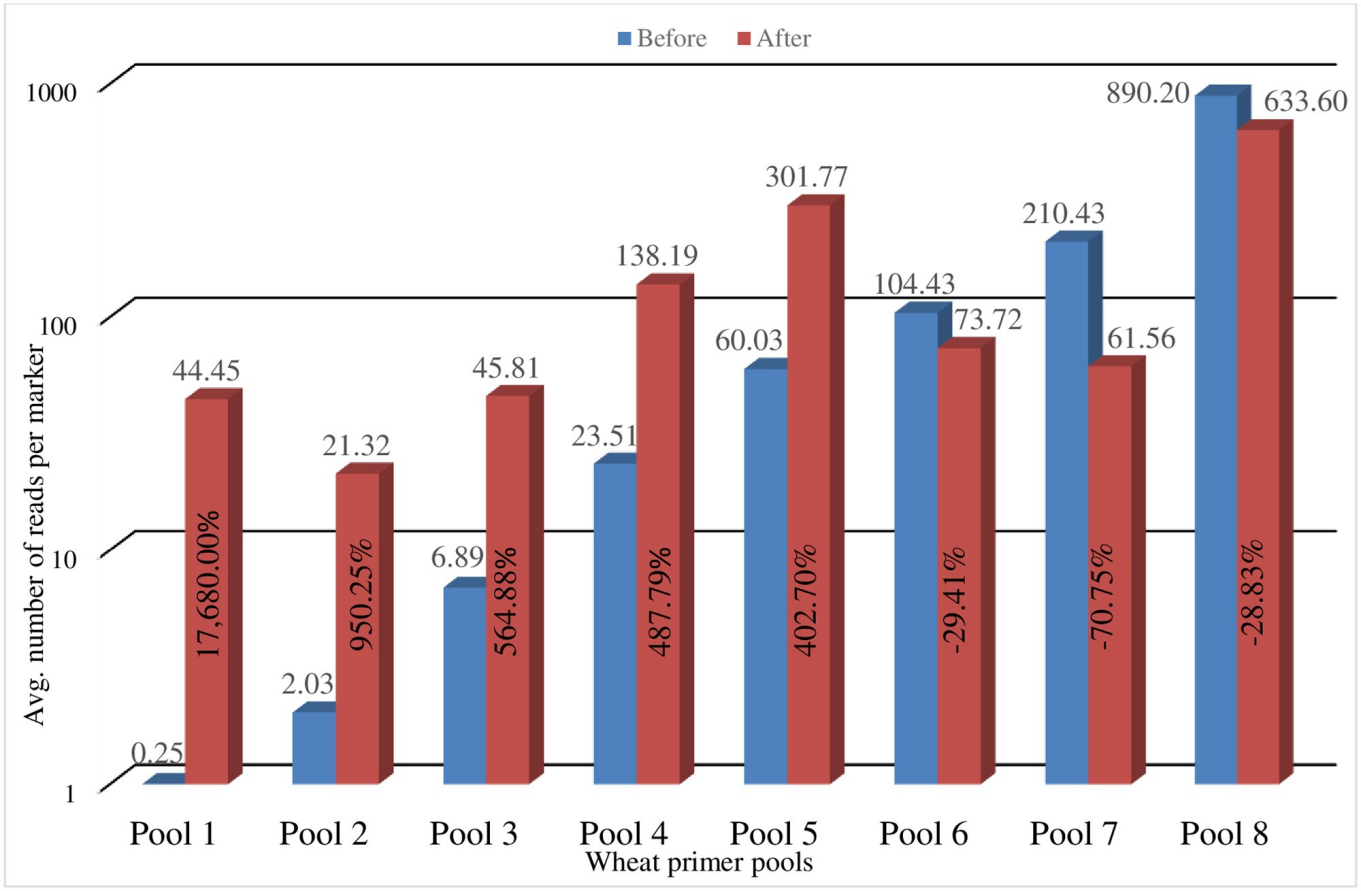
Before and after optimization of wheat primer pools. Depicts the average number of sequence reads per marker per pool before and after the reconstruction of each primer pool. The X axis shows the eight primer pools and the Y axis represents the number of sequence reads per marker per pool in log scale. Horizontal numbers show the average number of sequence reads generated per marker/sample. Vertical numbers indicate the percent increase or decrease in the average number of sequence reads generated per marker/sample after the primer pools were reconstructed.

The reconstructed primer pools were run with the two RIL populations and the DP. After analyzing the run data, reconstructed primer pool one averaged ~44 reads per marker/sample, primer pool 2 averaged ~22 reads per marker/sample, primer pool 3 averaged ~46 reads per marker/sample, primer pool 4 averaged ~138 reads per marker/sample and primer pool 5 averaged ~214 reads per marker/sample (Fig 2). Primer pools 1–5 showed a significant increase in the number of reads per marker/sample. Primer pools 6–8 showed a decrease in the number of reads per marker/sample as expected.

### Polymorphic information content (PIC) values

**Fig 3:**
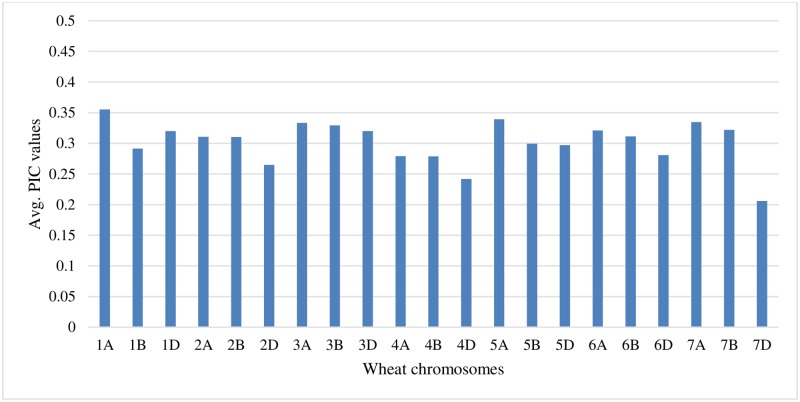
Average PIC values per chromosome. The Y axis depicts the average PIC values from the polymorphic SNP markers and the X axis shows the wheat chromosomes.

In order to calculate the PIC value of the current GMS SNP panel, the DP containing 96 cultivars from the Pacific Northwest (PNW) was genotyped. The PIC values were calculated according to Botstein et al. [27]. After data analysis, it was determined that the polymorphic SNP markers in this GMS protocol had an average PIC value of 0.30 out of a maximum value of 0.50 (Fig 3).

### Chinese spring nullisomic-tetrasomic analysis

Initially 270 markers were not able to be validated due to the amplification of paralogs or homoeoalleles. To resolve this issue these primers were made into a homoeoallele primer pool, then it and all other primer pools were run with the CS nulli-tetrasomic lines. This step was performed to determine the correct chromosomal location of amplified homoeoalleles or paralogs. After the nulli-tetrasomic lines data were analyzed, 53.3% or 144 primer pairs were validated in the homoeoallele primer pool. These markers amplified 155 homoeoalleles, 35 paralogs and each were assigned to correct chromosomal locations. The five wheat primer pools contained 336 primer pairs that amplified 241 homoeologous and 90 paralogous loci. Each were assigned to a chromosomal position utilizing the nulli-tetrasomic lines.

## Discussion

### GMS overview

Genotyping has become intertwined with NGS and SNPs have been adopted as the main marker type over the last decade. Multiple technologies have accelerated SNP discovery and have been utilized to create a more targeted approach for genotyping. GMS has shown that thousands of SNP markers can be highly multiplexed to genetically characterize a large number of samples in a single NGS run with high accuracy.

With 2,433 possible data points and a marker panel that averages a marker every 2.68 cM per chromosome, GMS can be used for most genetic studies, including genomic selection.

### Genetic distance between markers

The current SNP panel averages a marker every 2.68 cM but there are gaps on each chromosome that need to be addressed. Many of these gaps are in chromosomal regions which have proven difficult to design primers that could fill in these gaps. These chromosomal sections, such as 4D, contain sizeable gaps between markers that mapped in PNW material from the Illumina 90K SNP chip. For example, chromosome 4D which has the largest gap of 41.63 cM, all primers that were ordered and tested to fill in this gap failed to amplify products, leaving no more 90K SNP chip markers to test. This is true for much of the D genome, as the marker density is much lower when compared to the A and B genomes. The next step to reduce the cM distance between markers with large gaps is to mine genomic sequence data to find SNPs that could be developed into markers that could potentially fill in these troublesome gaps. For instance, the large gap on 4D is located between bases 3,396,702–5,593,858 on the 4D physical map. After mining this region, 12 new SNPs were found that could be used to close this large gap.

### Concordance of RIL data sets

The GMS and 90K SNP chip genotype data of the two RIL populations were in complete agreement except where the SNP chip results contained mis-clustered data points due to the co-hybridization of homoeoalleles. When co-hybridization occurred, homozygous samples were clustered in with the heterozygous samples. This happened when a homozygous sample also hybridized a homoeoallele that contained the SNPs alternate base, which resulted in the mis-clustering of datapoints. For instance, if a SNP is homozygous on chromosome 1A for sample one but also has an alternative allele on chromosome 1B, then it will be mis-clustered with heterozygotes in the 90K SNP chip results. When datapoints were mis-clustered, it was not possible to determine concordance between the 90K SNP and its GMS datapoint. This is a strength of GMS, as each primer has been tested for the amplification of homoeoalleles and if a SNP result is in question a researcher can view the sequence that was used to generate a SNP call to determine if a homoeoallele was amplified.

### Creating informative primer pools

In order to create a SNP panel that contained a majority of polymorphic markers, 90K SNP chip data were analyzed from the two RIL populations used in GMS testing. Markers which generated co-dominant results were selected for primer design and tested first. Once the polymorphic 90K SNP chip markers were exhausted, markers that generated monomorphic data were then used to fill in gaps on each chromosome. Even if these markers generated monomorphic data in PNW material, these markers may be polymorphic in material from different growing regions. Having preferentially selected polymorphic markers it was thought this would increase the overall averaged PIC values of the SNP panel, however, it did not. The averaged GMS marker panel PIC values were calculated to be 0.30, which was slightly higher than other calculations of the 90K SNP chip PIC values of 0.29 [28].

When the PIC values were calculated early in GMS research and development (R&D) the averaged PIC values was 0.39. This seemed to indicate that selecting polymorphic markers at the beginning of GMS R&D led to increased PIC values of the SNP panel. Once the polymorphic markers were exhausted and monomorphic markers were brought into the SNP panel to fill in gaps, this brought the overall PIC value down to 0.30. This averaged PIC values for the GMS marker panel remains in the relatively informative category which is typical for a bi-allelic SNP panel.

### Primer pool optimization

GMS primer pools were reconstructed according to the average number of sequence reads a marker generated per sample to determine if this reconstruction would increase this average. Grouping markers that had similar amplification rates in the same primer pool significantly increased the average number of sequence reads generated by primer pools 1–5 (Fig 2). The increased average number of sequence reads generated by each primer pool was likely due to two main factors: the competition between primer pairs was equalized and primer dimer formation was limited. The only parameter which changed in PCR #1 were the primer pools that had been reconstructed. This indicated that competition between primer pairs for reagents in PCR #1 had been normalized once primer pairs were grouped according to their rate of amplification. With limited primer dimer formation, primer pair performance improved. This allowed for a higher concentration of these markers to be available during PCR #1 to generate useful data and not occupy space on NGS chips or flow cells as primer dimer.

The doubling of barcode concentration in PCR #2 indicated that the reactions did not contain an adequate concentration of barcodes to label the majority of amplicons generated in PCR #1. When the barcode concentration was increased, the number of reads per marker increased by 32.4%. In a subsequent GMS test, the barcode concentration was tripled but data analysis showed no increase in the number of reads per marker. By doubling the barcode concentration in PCR #2, this concentration of barcodes was sufficient to ensure that most amplicon libraries were indexed.

### KIMs

The majority of KIMs were converted from KASP assays and many of those conversions to GMS proved to be difficult. Sequencing on NGS platforms limited amplicon sizes to 150–200 bp in high-throughput settings. GMS uses one marker panel for two NGS platforms which restricted the size of amplicons to 150 bp. This restricted amplicon size made primer design problematic for many SNPs, especially in regions with repetitive motifs and homopolymers. While designing primers for the Tamyb10-A1 KIM marker [29], it was discovered that upstream of the SNP there were four bases that matched at 100% but continuing upstream there was no sequence identity between the two alleles. A primer pair was successfully designed for the T allele; however, no successful primers have been designed for the C allele to date.

Another issue with converting KASP assays to GMS is many KASP makers generate short amplicons. Even with the incorporated adapters during PCR #1 and PCR #2, these amplicons risk being lost during size selection due to their short lengths. When adapting KASP assays to GMS, it is important to keep these parameters in mind.

### Template DNA in PCR #1

After the two RIL populations’ DNA extraction, quantification and robotic normalization, there was a large amount of variation in DNA concentrations from well to well. This variation could have been due to a substandard DNA extraction from individual wells, variation in the amount of tissue collected for extraction, degraded tissue, minimal tissue available for collection, or poor tissue disruption. The RIL sample that had the lowest DNA concentration averaged slightly less in the number of sequence reads generated per GMS run when compared to the sample which had the highest DNA concentration (Fig 1). After a regression analysis that observed the average number of sequence reads each RIL sample generated over five GMS runs, the R value was determined to be 0.0144. This indicated that there was minimal to no correlation between the amount of starting DNA template in PCR #1 and the average number of reads generated by each RIL sample per GMS run. This information specified that even when a sample’s DNA concentration is low it will likely generate high-quality GMS SNP results, which is important to know when template DNA is difficult to obtain.

### NGS library diversity

Sequence diversity of NGS libraries can be vital, especially when sequencing on an Illumina NGS platform. Sequencing libraries with low sequence diversity will result in runs that generate low-quality scores and suboptimal data output. This issue is remedied by spiking an Illumina run with a PhiX control library for sequence diversity, but this limits how much useful data a flow cell generates. The Ion Proton, which detects pH variances during the addition of nucleotides while sequencing, is not supposed to be affected by low sequence diversity. In GMS R&D, it was determined that when GMS libraries were not spiked with a diverse NGS library, the sequencing run generated less than 1,000,000 reads. It is difficult to speculate why this failure occurred. It is conceivable that since these libraries are only composed of 2,433 amplicons the sequencing solution intended to buffer each dimple could not handle this much uniformity of pH change across each chip which resulted in unusable reads. A GMS run was spiked with a diverse library and sequenced on an Ion Proton platform. After the resulting data was analyzed, it was found that this run generated high-quality reads with typical data output.

To achieve high-quality sequence data from both NGS platforms, each GMS run was initially spiked with 40% of a diverse library to ensure that each chip or flow cell had a balanced nucleotide load when sequencing. The resulting data were analyzed to ensure each GMS run had generated high-quality data and normal data output. Each subsequent GMS run was then loaded with a spike that was 5% less than the previous run and the data was again analyzed for high-quality reads. This was done for an additional eight runs and it was determined that a 5% spike of a diverse library was enough diversity to yield high-quality data from both the Ion Torrent and Illumina sequencing platforms.

### Assessment of open source high-density, high-throughput PCR genotyping methods

In the last decade multiple PCR-based genotyping technologies that use NGS have been developed including TAS, GT-seq, GBMAS in wheat, three-round multiplex PCR for SNP genotyping with NGS, and most recently, MTA-seq. The following is a comparison between these technologies: SNP calling accuracy ranged from 88% (TAS)– 99.9% (GT-seq) [4], with GMS at a 100% call accuracy. Amplicon lengths varied between 50bp (GT-seq)– 200bp (MTA-seq) [6], (TAS was excluded from this value as it was based on the defunct Roche 454 NGS platform) with GMS having a maximum amplicon length of 150bp. DNA template required for PCR ranged from 10ng (GT-seq,)– 50ng (MTA-seq), with GMS indicating samples with a total of 8ng for PCR template yielded similar reads per marker as those reactions with 200ng of template. The number of SNP markers per panel ranged from 19 markers (three-round multiplex PCR)– 443 markers (MTA-seq) with GBMAS in wheat at 33 markers, GT-seq at 192 markers, and GMS with 1,912 total markers. While each of these technologies could be useful in various genotyping studies, GMS has shown the capability of being sequenced on either an Illumina or Ion Torrent NGS platform, was able to capitalize on amplified homoeoalleles/paralogs as additional data points and has the densest SNP panel of these technologies.

### Homoeoallele amplification

One of the difficulties of genotyping polyploid organisms is the amplification or hybridization of homoeoalleles or paralogs. These off-target amplifications or hybridizations typically confound the resulting data. This is true for most genotyping technologies including Illumina SNP arrays to uniplex assays such as KASP. Hybridization of homoeoalleles in the Illumina 90K wheat array yields ambiguous results and ultimately leads to the loss of heterozygote samples in these instances. This makes the Illumina 90K SNP chip less useful as a genotyping tool for the genetic characterization of early generation material. GMS, by utilizing genetic tools such as the CS nulli-tetrasomic lines has been able to determine chromosomal positions of amplified off-target sequences. SNP markers that amplify homoeoalleles or paralogs in GMS introduce additional markers that make the platform more robust in some instances, as a single primer pair can track multiple SNPs.

## Conclusion

This study has presented a new PCR-based genotyping method that multiplexes thousands of SNP markers with high-throughput capabilities. Creating custom SNP panels to fit individual research needs can be done with ease, especially when exploiting publicly available SNP resources. The GMS protocol is simple, inexpensive, accurate, requires minimal bioinformatic knowledge and is applicable to most genetic studies, including genomic selection.

## Materials and methods

### DNA samples

Forty-eight individuals from each of two wheat RIL populations, the Louise/Penawawa [30] and the Coda/Brundage [31] and a 96-sample DP were genotyped in this study. These RIL populations were chosen as they had previously been genotyped with the 90K wheat Illumina Infinium Assay and represented both spring and winter wheat market classes. The DP contained a diverse selection of 96 cultivars from the PNW and was used to determine the PIC values of the current GMS marker set [27]. Genomic DNA was extracted using the LGC oKtopure platform and their proprietary sbedex plant nucleic acid extraction kit (LGC Genomics, Berlin, Germany). Extracted DNA was quantified using a Bio-Rad Fluorescent DNA Quantitation Kit (Bio-Rad Laboratories Inc., Hercules, CA), read on a BioTek Synergy 2 plate reader (BioTek, Winooski, VT) and then normalized to a concentration of 20 ng/μL using a QIAgility liquid handler (Qiagen, Hilden Germany).

### Primer design

The majority of SNP primer pairs were designed using sequences from the wheat Illumina Infinium 90K SNP chip array [32]. SNPs were selected evenly across each chromosome with no more than a 2 cM gap between each and their sequences were input into the Sequenom MassArray Assay Design 4.0 software (Sequenom, San Diego, CA) for primer design. Primer design parameters were set with the minimum sequence length at 125 bp, the optimal sequence length at 135 bp and the maximum length set at 150 bp with an ideal melting temperature between 56°C—60°C. After PCR #1 primers were designed a M13 tail [33] was synthesized to the 5’ end of each forward primer and the P1/B sequence (Life Technologies, Carlsbad, CA), to the 5’ end of each reverse primer for sequencing on the Ion Proton. The i5 adapter sequence was synthesized to the 5’ end of each forward primer and the i7 adapter sequence was synthesized to the 5’ end of each reverse primer for sequencing on an Illumina NGS platform (Illumina, San Diego, CA). These adapters resulted in primers that averaged 42 bp in length for the Ion Proton and 53bp for Illumina primers (S1 Table). PCR #2 determined which NGS platform the GMS libraries would be sequenced on and uniquely identified each well with an index\barcode through amplification of the PCR #1 product (S1 Fig). The 5’ end of each PCR #2 primer started with a platform specific adapter sequence. The Ion Torrent primers contained the Ion A/P1B adapter sequences and the Illumina primers started with index 2/index 1 read adapter sequences. This was followed by a unique eight base barcode and then the amplicon sequence adapter. For the Ion Proton each PCR #2 primer ended with the M13 or P1B sequence and the Illumina primers ended with the i5 or i7 sequence adapters (S2 Table).

Once primer pairs had been ordered, GMS key files were generated for data analysis. A key file is a text file that is in FASTA format and contains the exact sequence that each marker should amplify (S3 Table). The first line in this file is the marker name and is followed on the next line by its amplicon sequence. This process of generating a key file is done for all markers in a GMS run. Each marker’s key file sequence contained the ambiguity code for SNP calling and were what sequence reads were aligned with in the bioinformatic pipeline to generate SNP calls.

In addition to the SNPs that were selected from across the three wheat genomes, 58 agronomic, quality, or disease resistance SNP markers were chosen for their importance in wheat breeding/improvement. The KIMs were derived from other genotyping assays including KASP [15], CAPS, and insertions/deletions (INDELs). See S4 Table for a comprehensive KIM list.

### Library construction

#### PCR #1

Library production followed protocols as described by the Schnable lab and Bernardo et al. with various alterations [5,10]. The master mix for a single reaction in PCR #1 was as follows: 1X MCLAB Taq PCR Buffer with 20 mM MgCl_2_ (MCLAB, San Francisco, CA), an additional 1.125 mM MgCl_2_, 500 μM of each dNTP, primer pool at 12.5 nM, 1 unit HoTaq DNA Polymerase (MCLAB, San Francisco, CA), and 80 ng template DNA for a total volume of 10 μL. Thermocycler conditions for PCR 1: denaturing was performed for 10 minutes at 94°C, followed by 35 cycles of 20 seconds at 94°C, 2 minutes at 56°C, 30 seconds at 68°C, and the last elongation was performed for 3 minutes at 72°C. If multiple primer pools were employed during PCR #1, an equal volume from each PCR #1 plate was pooled into a corresponding single 96-well plate. Once all PCR #1 plates were pooled in a single plate, an equal volume of molecular grade water was added to each well for a 1:1 dilution. This dilution served as template in PCR #2.

#### PCR #2

The master mix for a single reaction in PCR #2 was as follows: 1X MCLAB Taq PCR Buffer with 20 mM MgCl_2_ (MCLAB, San Francisco, CA), an additional 1.125 mM MgCl_2_, 500 μM of each dNTP, P1/B reverse primer at 0.4 μM, 1 unit HoTaq DNA Polymerase (MCLAB, San Francisco, CA) and 0.8 μM of unique barcodes in a reaction volume of 5 μL. The thermocycler conditions for PCR #2 were as follows: denaturing was performed for 10 minutes at 94°C, followed by 15 cycles of 20 seconds at 94°C, 30 seconds at 60°C, 1 minute at 72°C, and the last elongation was performed for 3 minutes at 72°C. Once PCR #2 was completed, an equal volume from each well was pooled into a 1.5 mL Eppendorf tube.

### Sample purification, size selection, quantification, and sequencing

After the PCR steps were completed, each pool was purified using a QIAquick PCR Purification Kit (Qiagen, Hilden Germany) per product instruction. To keep the pool(s) as concentrated as possible the elution was performed with 30 μL elution buffer (EB). The pool(s) were further purified using Agencourt AMPure XP beads (Beckman Coulter, Indianapolis, IN) using 0.8 μL of beads per 1 μL of sample. The first size selection was done on a 4% E-Gel SizeSelect Gel (Life Technologies, Carlsbad, CA) and PCR products were excised between 140 bp and 250 bp. Gel purification was performed with a QIAEX II Gel Extraction Kit (Qiagen) and the elution was done again with 30 μL EB to keep the pool(s) concentrated as possible. The final size selection was done with a 2% E-Gel II Size Select Gel (Life Technologies, Carlsbad, CA) collecting between 140 bp to 250 bp, followed by the final PCR purification with the Qiagen PCR Purification Kit. Quantification of the pooled GMS libraries were performed using the Qubit dsDNA HS assay kit (Life Technologies, Carlsbad, CA) and library quality was assessed using an Agilent DNA 7500 Kit (Agilent, Santa Clara, CA). To pass quality control the libraries must have a minimum concentration of 80 pmol/L and have a size distribution between 185 bp to 260 bp. The diluted sample libraries were then loaded onto an Ion Chef (Life Technologies, Carlsbad, CA) for the remainder of library preparation. Once the Ion Chef finished, the loaded chips were sequenced on an Ion Torrent Proton NGS platform (Life Technologies, Carlsbad, CA). The reagents used for the last library step on the Ion Chef and Ion Proton were the Ion PI^™^ Hi-Q Sequencing 200 and the Ion PI^™^ Chip Kit v3. Each GMS run is spiked with 5% of a diverse NGS library compatible with the Ion Proton to increase sequence diversity.

### Bioinformatic pipeline

In the first step of this pipeline, sequence reads were aligned against each other at a 100% match using the program cd-hit-est to generate “seeds” [34]. These seeds were then aligned to GMS key files at a 100% match using the program Clustal Omega [35]. This script then determined the similarity of the seeds to the key files excluding the SNP of interest and discarded seeds that were less than a 100% match to the key files. In addition, if a seed did not represent at least 10% of the total read count for the key files they were discarded. The remaining seeds were again aligned to the GMS key files using *Muscle* [36]. The final Perl script generated the SNP and hit count reports and also generated multiple sequence alignment FASTA files.

### Pipeline output

The results are in tab delimited text format with one file containing the individual SNP results and another file with the number of sequence reads per marker per sample. The pipeline also generates multiple sequence alignment FASTA files for each marker run with each sample. These FASTA files allow the researcher to see the exact sequence that was used to generate the SNP calls for each marker per sample.

### Chinese spring nullisomic-tetrasomic lines analysis

The Chinese Spring nullisomic-tetrasomic lines [37] were used to determine the chromosomal locations of amplified homoeoalleles and paralogs by GMS markers. The nulli-tetrasomic lines were used for this analysis as each line lacks a single wheat chromosome. A homoeoallele primer pool was constructed from markers which only amplified homoeoalleles or paralogs that had been previously thrown out of the GMS SNP panel. To bring as many of these markers back into the marker panel they were run with the nulli-tetrasomic lines. The resulting SNP report was generated at a 95% sequence match between the GMS key files and seeds. This was done to include as many amplified homoeoalleles and paralogs into the SNP report. Multiple sequence alignment FASTA files from numerous samples were analyzed for each marker in the homoeoallele primer pool. These files were searched for seeds that did not match the key file sequence at 100%. In most instances at least two seeds aligned to a given GMS key file, one seed aligned at 100% sequence match and the other aligned at less than 100%. The seed sequence that did not match at 100% was used to search the nulli-tetrasomic lines sequence results. As each individual nulli-tetrasomic line is without a specific chromosome, the location of homoeoalleles and paralogs could be inferred when a searched sequence was not found from a specific nulli-tetrasomic line while this searched sequence was found in all other lines. This process was repeated for each marker in the homoeoallele primer pool and then with the 5 wheat primer pools as well. This analysis made it possible to determine which chromosome a homoeoallele or paralog was amplified from and were incorporated into the GMS SNP results.

## Supporting information

S1 Table Primer pairs with primer pool id and adapter sequences. (XLSX)

S2 Table Ion Torrent and Illumina barcodes/indexes. (XLSX)

S3 Table GMS key file for data analysis. (XLSX)

S4 Table Full list of KIMs in GMS and pertinent information about each marker. (XLSX)

S1 Fig GMS Illumina library amplification steps. (DOCX)
