# Supplementary material for: Genotyping by multiplexed sequencing (GMS): A customizable platform for genomic selection
Source: PLoS One. 2020 May 1;15(5):e0229207. doi: 10.1371/journal.pone.0229207 (PMC7194356; doi:10.1371/journal.pone.0229207)
Supplement: S1 Fig — (DOCX) [file pone.0229207.s005.docx]

**PCR#1**

Forward locus specific primer (LSP)

i5 adapter

TCGTCGGCAGCGTCAGATGTGTATAAGAGACAGNNNNNNNNNNNNNNNNNNXXXXX…..XXXXXNNNNNNNNNNNNNNNNNNGACAGAGAATATGTGTAGAGGCTCGGGTGCTCTG

Target sequence

AGCAGCCGTCGCAGTCTACACATATTCTCTGTCNNNNNNNNNNNNNNNNNNXXXXX…..XXXXXNNNNNNNNNNNNNNNNNNCTGTCTCTTATACACATCTCCGAGCCCACGAGAC

Reverse locus specific primer (LSP)

i7 adapter

**PCR #2**

Index 1 read seq

i7 adapter

Index

TCGTCGGCAGCGTCAGATGTGTATAAGAGACAGNNNNNNXX…..XXNNNNNNGACAGAGAATATGTGTAGAGGCTCGGGTGCTCTG
 CTGTCTCTTATACACATCTCCGAGCCCACGAGAC--------ATCTCGTATGCCGTCTTCTGCTTG

Index 2 read seq

i5 adapter

AATGATACGGCGACCACCGAGATCTACACTCGTCGGCAGCGTCAGATGTGTATAAGAGACAG

AGCAGCCGTCGCAGTCTACACATATTCTCTGTCNNNNNNXX…..XXNNNNNNNCTGTCTCTTATACACATCTCCGAGCCCACGAGAC

**Final library**

LSP

Index 2 read seq

i5 adapter

AATGATACGGCGACCACCGAGATCTACACTCGTCGGCAGCGTCAGATGTGTATAAGAGACAGNNNNNNXX…..XXNNNNNNNGACAGAGAATATGTGTAGAGGCTCGGGTGCTCTG--------TAGAGCATACGGCAGAAGACGAAC
TTACTATGCCGCTGGTGGCTCTAGATGTGAGCAGCCGTCGCAGTCTACACATATTCTCTGTCNNNNNNXX…..XXNNNNNNNCTGTCTCTTATACACATCTCCGAGCCCACGAGAC--------ATCTCGTATGCCGTCTTCTGCTTG

Index

LSP

Index 1 read seq

i7 adapter

**S1 Fig.** **GMS Illumina library amplification steps.** PCR#1 amplified targeted SNPs using locus specific primer pairs. Each forward primer had the Illumina i5 adapter sequence synthesized to the 5’ end and the reverse primer had the Illumina i7 adapter sequence on its 5’ end. During PCR#2 NGS platform specific adapters were added along with a unique index/barcode. The forward primer consisted of the index 2 read adapter followed by the i5 adapter sequence. The reverse primer consisted of the index 1 read sequence, followed by a unique index/barcode and ends with the i7 adapter sequence. See Bernardo et al. (10) for the Ion Proton library construction.
